# Transcriptome-wide m^6^A methylome analysis uncovered the changes of m^6^A modification in oral pre-malignant cells compared with normal oral epithelial cells

**DOI:** 10.3389/fonc.2022.939449

**Published:** 2022-09-28

**Authors:** Xun Chen, Liutao Chen, Yuquan Tang, Yi He, Kuangwu Pan, Linyu Yuan, Weihong Xie, Shangwu Chen, Wei Zhao, Dongsheng Yu

**Affiliations:** ^1^ Hospital of Stomatology, Guanghua School of Stomatology, Guangdong Provincial Key Laboratory of Stomatology, Sun Yat-sen University, Guangzhou, China; ^2^ Guangdong Key Laboratory of Pharmaceutical Functional Genes, Department of Biochemistry, School of Life Sciences, Sun Yat-sen University, Guangzhou, China; ^3^ State Key Laboratory for Biocontrol, Department of Biochemistry, School of Life Sciences, Sun Yat-sen University, Guangzhou, China

**Keywords:** m^6^A modification, m^6^A regulatory genes, MeRIP sequencing, precancerous cells, dysplastic oral keratinocyte (DOK), human oral epithelial cell (HOEC), oral squamous cell carcinoma cell

## Abstract

As the most common post-transcriptional RNA modification, m^6^A methylation extensively regulates the structure and function of RNA. The dynamic and reversible modification of m^6^A is coordinated by m^6^A writers and erasers. m^6^A reader proteins recognize m^6^A modification on RNA, mediating different downstream biological functions. mRNA m^6^A modification and its corresponding regulators play an important role in cancers, but its characteristics in the precancerous stage are still unclear. In this study, we used oral precancerous DOK cells as a model to explore the characteristics of transcriptome-wide m^6^A modification and major m^6^A regulator expression in the precancerous stage compared with normal oral epithelial cell HOEC and oral cancer cell SCC-9 through MeRIP-seq and RT-PCR. Compared with HOEC cells, we found 1180 hyper-methylated and 1606 hypo-methylated m^6^A peaks and 354 differentially expressed mRNAs with differential m^6^A peaks in DOK cells. Although the change of m^6^A modification in DOK cells was less than that in SCC-9 cells, mRNAs with differential m^6^A in both cell lines were enriched into many identical GO terms and KEGG pathways. Among the 20 known m^6^A regulatory genes, FTO, ALKBH5, METTL3 and VIRMA were upregulated or downregulated in DOK cells, and the expression levels of 10 genes such as METTL14/16, FTO and IGF2BP2/3 were significantly changed in SCC-9 cells. Our data suggest that precancerous cells showed, to some extent, changes of m^6^A modification. Identifying some key m^6^A targets and corresponding regulators in precancerous stage may provide potential intervention targets for the prevention of cancer development through epigenetic modification in the future.

## Introduction

N^6^-methyladenine (m^6^A) methylation is a dynamic and reversible modification regulated by methyltransferases and demethylases in different types of RNAs including messenger RNA (mRNA). This is the most prevalent post-transcriptional regulatory markers on eukaryotic RNAs and one third of the total mammalian mRNA has 3-5 m^6^A modifications in each mRNA (1, 2). Most m^6^A sites are located in the conserved motif DRACH (D=G/A/U, R=G/A, H=A/U/C), which are usually found in 3′ UTRs and near stop codons in mRNAs (1, 2).

m^6^A is decorated by m^6^A methyltransferase complex, and its components include METTL3/14/16, WTAP, RBM15, RBM15B, VIRMA, and ZC3H13. They are collectively referred to as m^6^A writers. On the contrary, demethylases such as FTO, ALKBH5 and ALKBH3 can remove m^6^A and act as erasers. The cooperation of m^6^A writers and erasers regulates the dynamic and reversible modification of m^6^A. m^6^A modification of RNAs must be recognized by m^6^A reader proteins to mediate different downstream biological functions. Several categories of proteins function as m^6^A reader, including YT521-B homology (YTH) domain-containing proteins YTHDC1/2 and YTHDF1/2/3, IGF2 mRNA binding proteins IGF2BP1/2/3, heterogeneous nuclear ribonucleoproteins HNRNPA2B1 and HNRNPC, and eukaryotic initiation factor 3 (eIF3). RNA m^6^A modification plays an important role in regulating RNA splicing, translation, stability, translocation, and advanced structure (3, 4).

There is increasing evidence that RNA m^6^A methylation and its regulators are associated with a variety of human diseases, especially cancer (3–5). m^6^A regulators may act as writers to catalyze or erasers to remove m^6^A modifications in the mRNAs of oncogenes and tumor suppressor genes, which are then recognized by readers to regulate the expression of these genes and their downstream biological functions. The role of m^6^A modification involves many aspects of tumors. For example, demethylase FTO-mediated m^6^A demethylation of cytidine deaminase APOBEC3B mRNA promotes arsenic-induced mutagenesis (6). FTO promotes growth and metastasis of gastric cancer through m^6^A demethylation of caveolin-1 and metabolic regulation of mitochondrial dynamics (7). FTO-mediated downregulation of DACT1 mRNA stability promotes Wnt signaling to facilitate osteosarcoma progression (8). Downregulation of FTO promotes EMT-mediated progression of epithelial tumors and sensitivity to Wnt inhibitors (9). FTO promotes hepatocellular carcinoma tumorigenesis through mediating PKM2 demethylation (10). m^6^A writer METTL3 promotes chemo- and radioresistance in pancreatic cancer cells (11). METTL3 stabilizes HK2 and SLC2A1 (GLUT1) expression in colorectal cancer and m^6^A-dependent glycolysis enhances colorectal cancer progression (12). METTL3 promotes tumor development by decreasing APC gene expression mediated by APC mRNA m^6^A-dependent YTHDF binding (13).

The significance of m^6^A modification in head and neck squamous cell carcinoma (HNSCC) has been well reviewed (4). m^6^A plays an important role in the tumorigenesis, drug resistance and prognosis of oral squamous cell carcinoma (OSCC). Methyltransferase-like 3 (METTL3) promotes OSCC by regulating m^6^A of p38 (14), BMI1 (15), c-Myc (16), PRMT5 and PD-L1 (17). Bioinformatics analysis reveals that HNRNPC and HNRNPA2B1 facilitate progression of OSCC *via* EMT (18, 19). m^6^A demethylase fat mass and obesity-associated protein (FTO) plays an oncogenic role in arecoline-induced OSCC progression (20) and regulates autophagy and tumorigenesis by targeting eukaryotic translation initiation factor gamma 1 (eIF4G1) in OSCC (21). DEAD-box helicase 3 X-linked (DDX3) is a human RNA helicase that directly regulates m^6^A demethylase ALKBH5, thereby reducing m^6^A methylation of cancer stem cell transcription factor fork head box protein M1 (FOXM1) and Nanog, leading to chemoresistance (22). Analysis based on the cancer genome atlas (TCGA) data indicates that m^6^A RNA methylation regulators can predict the prognosis of patients with HNSCC (23).

Importantly, m^6^A in cancer seems to function as a double-edged sword. The methylation of some genes is related to tumorigenesis, while the demethylation of others will promote tumorigenesis (24, 25). A single m^6^A regulator can exert biological function *via* different target genes in the same cancer (26, 27). As described above, the same m^6^A regulator may act different functions in different tumors (6–10). These findings demonstrated that regulatory networks of m^6^A methylation in cancers are extremely complex and need to be further explored.

Although m^6^A modification together with corresponding regulators was involved in the occurrence and progression of a variety of cancers, the characteristics of this epigenetic modification in the precancerous stage are still unclear. A few of studies have attempted to explore the m^6^A modification in premalignant stage. When studying the role of m^6^A regulatory genes in colorectal carcinogenesis, it was found that the expression of YTHDF1, IGF2BP1, IGF2BP3, and EIF3B in adenoma was up-regulated at the protein level (28). Oral submucosal fibrosis (OSF) is a precancerous condition. Compared with normal tissues, the overall m^6^A level in OSF tissues was increased, suggesting that m^6^A modification contributes to OSF (29). However, to our knowledge, the change of m^6^A profile and the expression of m^6^A regulators in precancerous cells have not been well studied. In this study, we analyzed the transcriptome-wide m^6^A methylome of oral precancerous DOK cells (30) by MeRIP-seq and RNA-seq, determined the expression of major m^6^A regulators of these cells by RT-PCR, and compared their m^6^A modification with that of normal oral epithelial cells and oral cancer cells.

## Materials and methods

### Cells and cell culture

This study involved three cell lines, including immortalized normal human oral epithelial cell (HOEC) (BNCC340217) provided by BeNa Culture Collection (Bnbio, Beijing, China), human dysplastic oral keratinocyte (DOK) and oral squamous cell carcinoma cell SCC-9 provided by Shanghai Guandao Biological Engineering Co., Ltd (Sgdbio, Shanghai, China) (30, 31). DOK cells were established from human dysplastic oral mucosa and were considered to be precancerous cells (30). DOK cells were maintained in RPMI-1640 medium, HOEC and SCC-9 cells were maintained in DMEM medium, supplemented with 10% FBS (GIBCO, Australia), 100 U/mL penicillin G and 100 μg/mL streptomycin, in a humidified atmosphere of 5% CO_2_ at 37°C. About 5×10^7^ cells were harvested for MeRIP-seq and RNA-seq.

### MeRIP sequencing and RNA sequencing

Transcriptome-wide m^6^A sequencing was performed as described previously (32, 33). Briefly, total RNA was isolated using TRIzol reagent (Invitrogen, Carlsbad, CA, USA) and quantified. RNA integrity was evaluated by Bioanalyzer 2100 (Agilent, CA, USA). Poly (A) RNA was purified using Dynabeads Oligo (dT)25-61005 (Thermo Fisher, CA, USA) and fragmented into about 100nt using Magnesium RNA Fragmentation Module (NEB, USA). The RNA fragments were divided into two portions, one was kept as input and the other was used to enrich m^6^A-methylated RNA fragments by immunoprecipitation with m^6^A-specific antibody (Synaptic Systems, Germany). RNA-seq library preparation included double stranded cDNA synthesis, addition of A-tailing, adapter ligation, amplification of ligated products, and library purification. The paired-end sequencing (PE150) of libraries was performed on Illumina Novaseq™ 6000 platform (LC-Bio Technology CO., Ltd., Hangzhou, China), following the manufacturer’s protocol.

### Bioinformatics analysis

The fastp tool (https://github.com/OpenGene/fastp) was used to remove the low quality reads and trim adaptors (34). Sequence quality of IP and input samples was verified using FastQC (https://www.bioinformatics.babraham.ac.uk/projects/fastqc/) and RseQC (http://rseqc.sourceforge.net/) (35, 36). Clean reads were then mapped to the reference genome Homo sapiens (Version: v101) using HISAT2 (http://daehwankimlab.github.io/hisat2) (37). m^6^A peak calling and analysis of differentially methylated peaks were performed by R package exomePeak2 (https://bioconductor.org/packages/release/bioc/html/exomePeak2.html) (38), and peaks were annotated by intersection with gene architecture using R package ANNOVAR (http://www.openbioinformatics.org/annovar/) (39). The MEME (http://meme-suite.org) (40) and HOMER (http://homer.ucsd.edu/homer/motif) were used for *de novo* and known motif finding, followed by localization of the motif with respect to peak summit. The expression level of all transcripts and genes from input libraries was analyzed through calculating FPKM (total exon fragments/mapped reads (millions) × exon length (kb)) using StringTie (https://ccb.jhu.edu/software/stringtie) (41). The R package edgeR (https://bioconductor.org/packages/edgeR) (42) was used to identify differentially expressed transcripts and genes, and the threshold was set to |log_2_fold change (FC)|≥1 and *p* value < 0.05. The differentially expressed genes and differentially methylated coding genes were subjected to Gene Oncology (GO) functional enrichment analysis and Kyoto Encyclopedia of Genes and Genomes (KEGG) pathway analysis (43).

### RNA extraction and quantitative real-time RT-PCR

Total RNA was isolated by incubating the cells in a 25-cm^2^ culture flask with 1 mL TRIzol reagent (Invitrogen Life Technologies, Carlsbad, CA, USA) in accordance with the manufacturer’s instructions. After genomic DNA was removed by treatment with gDNA Eraser, RNAs were reverse-transcribed with random hexamer primers using PrimeScript RT Enzyme Mix I (PrimeScriptTM RT reagent Kit with gDNA Eraser, TaKaRa, Shiga, Japan). Real-time PCR were processed in triplicate and finished in 20 μL reaction volumes with SYBR^®^ Premix Ex Taq II (Tli RNaseH Plus, TaKaRa, Shiga, Japan) and the ABI PRISM^®^7900 system (ABI). The threshold cycles and relative fold differences were calculated with 2^-ΔΔCt^. The primers used in the study are listed in **Table S1** and proved to be effective in previous studies (44, 45).

### Statistical analysis

For MeRIP-seq, aligned reads were used for peak calling of the MeRIP regions, and significantly enriched regions (peaks) were determined at a threshold of log_2_FC ≥ 1 and *p* value < 0.05. For MeRIP sequencing and RNA sequencing data, differentially methylated peaks were identified through exomePeak2 (38) and differentially expressed genes were identified by the edgeR in R package (42) according to the criteria |log_2_FC| ≥ 1 and *p* < 0.05. The statistical analysis of RT-PCR data were done using Graphpad Prism 8, and *p* value were calculated using two tailed unpaired student’s t-test. * represent *p* < 0.05 and ** represent *p* < 0.01.

## Results

### Overall features of m^6^A methylation in different oral cells

In order to explore the characteristics of m^6^A methylation in precancerous cells, we did MeRIP-seq and RNA-seq of three oral cell lines, including normal human oral epithelial cell HOEC, precancerous dysplastic oral keratinocyte DOK, and oral squamous cell carcinoma cell SCC-9. Sequencing data were summarized in **Table S2**, and the reads containing adaptor, low quality bases and undetermined bases were removed from raw data to generate clean data. More than 90% valid data from IP and input samples can be mapped to exons of genes in the reference genome.

**Figure 1:**
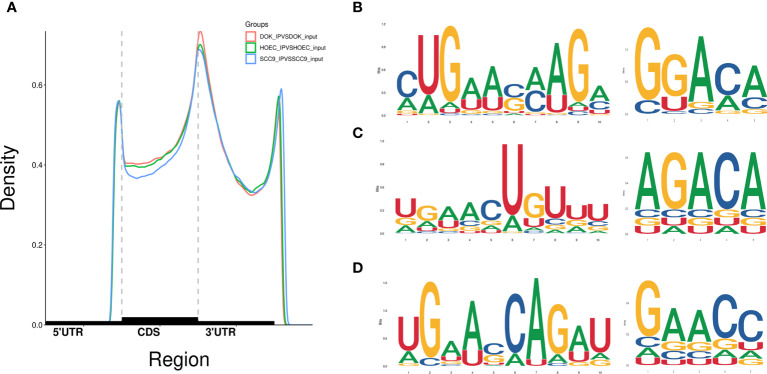
Distribution of m^6^A peaks across the length of mRNA transcripts and the representative m^6^A motifs with typical conserved sequence. **(A)** The m^6^A peaks were highly enriched in 3`UTR and stop codon regions. **(B–D)** The m^6^A motifs with typical conserved sequence in HOEC, DOK and SCC-9 cells, respectively.

Based on clean data, a total of 43,296, 41,946 and 41,817 m^6^A peaks were identified in HOEC, DOK and SCC-9 cells, respectively. In all three cell lines, m^6^A peaks were highly enriched in 3`UTR and stop codon regions, and their distribution and density across the length of mRNA transcripts were similar (**Figure 1A**). Some enriched m^6^A peaks have typical conserved motifs (**Figures 1B–D**).

### m^6^A profile changed in oral pre-malignant cells

**Figure 2:**
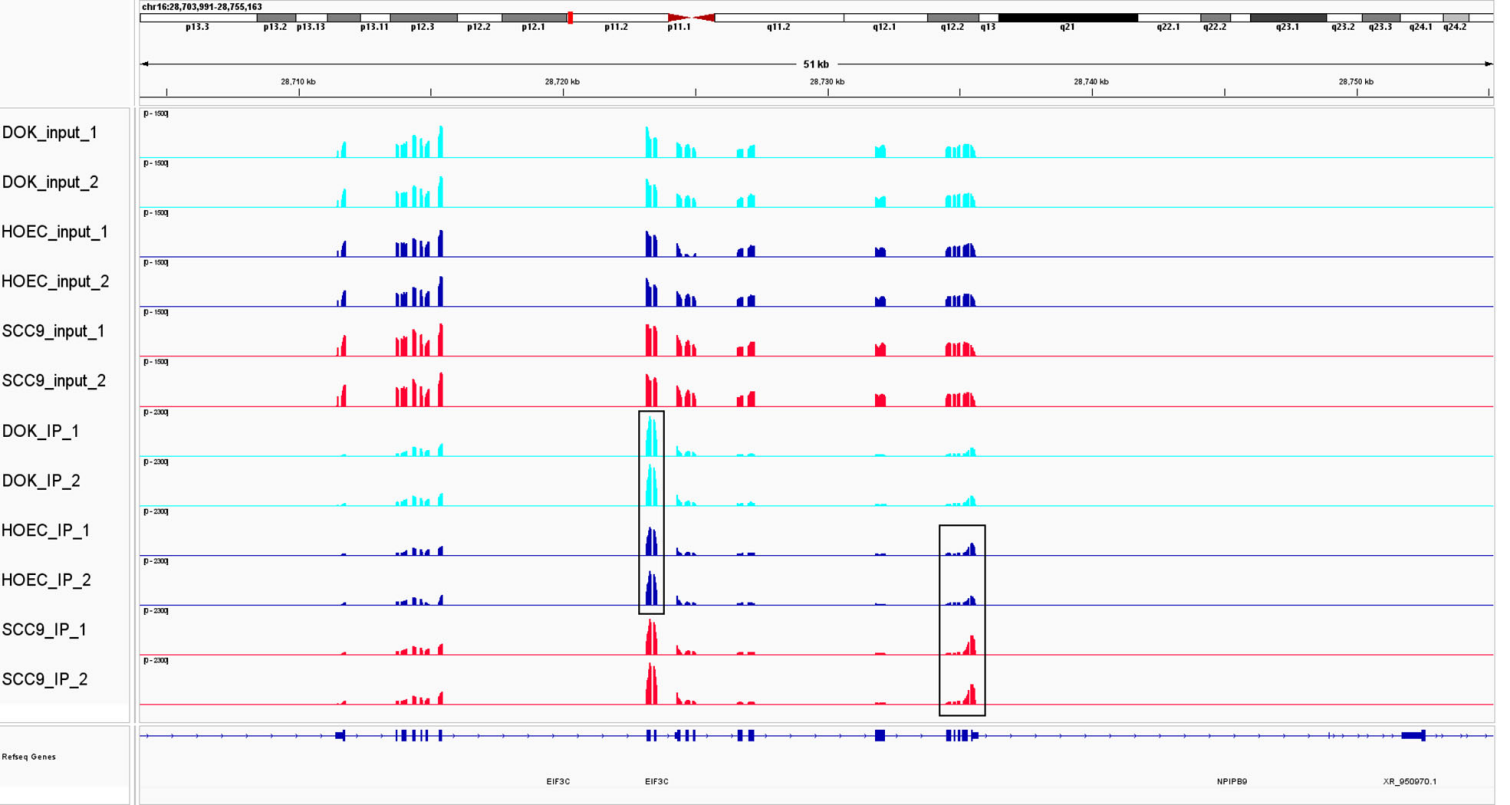
The change of m^6^A modification in DOK and SCC-9 cells compared with HOEC cells. m^6^A peak clusters from three cells are shown along the length of the EIF3C transcript. Compared with HOEC cells, hyper-methylated peaks were observed in exonic region in DOK cells and in 3`UTR in SCC-9 cells. EIF3C, eukaryotic translation initiation factor 3 subunit C.

When analyzing the differential m^6^A peaks between each two cell lines, it was found that compared with HOEC cells, there were 1,180 hyper-methylated and 1,606 hypo-methylated m^6^A peaks in DOK cells and 3,916 hyper-methylated and 1,349 hypo-methylated m^6^A peaks in SCC-9 cells (|log_2_FC|≥1.0 and *p* < 0.05). Compared with DOK cells, 5,120 hyper-methylated and 1,243 hypo-methylated m^6^A peaks were identified in SCC-9 cells. Obviously, the m^6^A modification in DOK cells has changed in general compared with HOEC cells, but these changes seem to be insufficient compared with those in SCC-9 cells. Compared with HOEC cells, the top 20 genes with the most significant m^6^A peak changes in DOK and SCC-9 cells were shown in **Table 1** and the m^6^A peak changes of a representative gene were shown in **Figure 2**. These genes in DOK cells were also inconsistent with those in SCC-9 cells, further indicating that the modification of m^6^A in the early phase of carcinogenesis has its own characteristics.

**Table 1:** The top 20 altered m^6^A peaks in DOK and SCC-9 cells compared with HOEC cells.

DOK				SCC-9			
Hyper-methylated		Hypo-methylated		Hyper-methylated		Hypo-methylated	
Genes	Peak region	Genes	Peak region	Genes	Peak region	Genes	Peak region
EIF3C	exonic	SULT1A4	UTR3	SLC9A3	exonic	SLX1B	UTR3
NOMO1	exonic	NOMO2	UTR3	SRCIN1	UTR5	AC148477	ncRNA_exonic
TMLHE	UTR3	FAM72D	UTR3	SERF1B	UTR3	PHTF1	intronic
TBC1D3D	exonic	F8A1	UTR5	AL513218	ncRNA_exonic	HSPA1B	exonic
ETS1	UTR3	HES2	UTR3	SCAMP1	UTR5	SMG1P5	ncRNA_exonic
VGF	exonic	ZNF10	UTR3	PWP2	exonic	SP140L	intronic
ROR1	UTR3	SLC30A4	UTR3	NCK1-DT	ncRNA_exonic	ZNF467	UTR5
MAGEA9B	UTR5	LINC02341	ncRNA_exonic	FAM169B	ncRNA_exonic	BRSK1	UTR3
CD177	UTR3	C2orf42	UTR5	EIF3C	UTR3	STEAP2	UTR3
TTC28-AS1	ncRNA_exonic	CYTH3	exonic	MIR137HG	ncRNA_exonic	ITPR1	UTR3

UTR3, 3’ untranslated region; UTR5, 5’ untranslated region.

**Figure 3:**
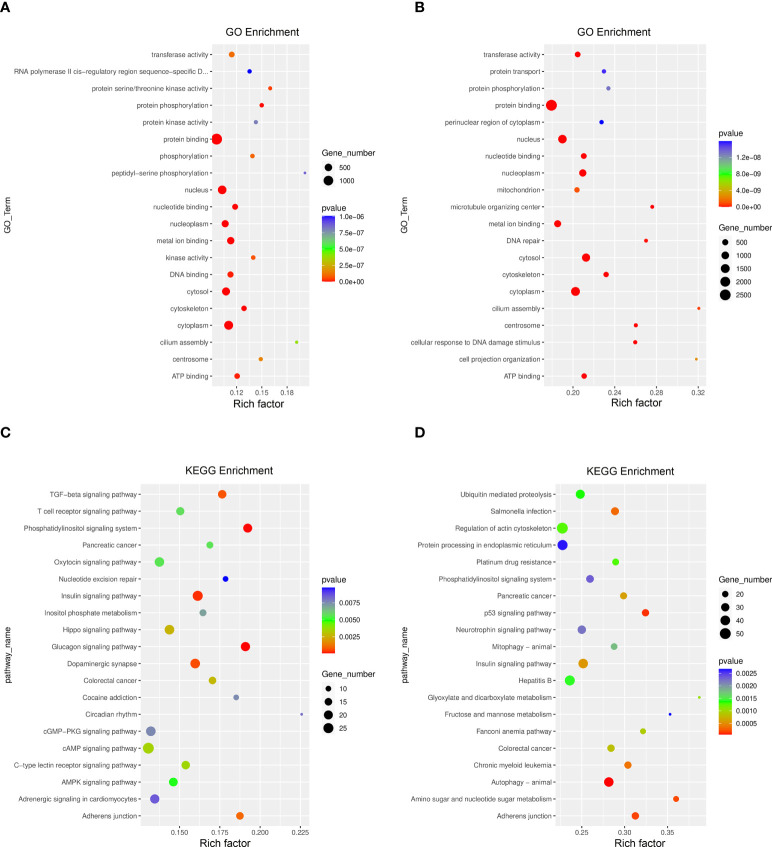
GO function and KEGG pathway enrichment of differentially methylated mRNA. **(A, B)** Compared with HOEC cells, top 20 significantly enriched GO terms in DOK and SCC-9 cells, respectively. **(C, D)** Compared with HOEC cells, top 20 significantly enriched KEGG pathways in DOK and SCC-9 cells, respectively.

The GO function and KEGG pathway enrichment of differentially methylated mRNAs were analyzed to explore the biological significance of m^6^A modification. Among the top 20 GO terms enriched with differentially methylated mRNAs between DOK and HOEC cells, 13 terms are consistent with those between SCC-9 and HOEC cells (**Figures 3A, B**). Those differentially methylated genes were significantly enriched in some important biological processes, such as transferase activity, protein phosphorylation, and protein binding and so on. In the first 20 enriched KEGG pathways of differentially methylated mRNAs between DOK and HOEC cells, 5 pathways were also enriched between SCC-9 and HOEC cells, including phosphatidylinositol signaling system, pancreatic cancer, insulin signaling pathway, colorectal cancer and adherens junction (**Figures 3C, D**). The above data may suggest that precancerous DOK cells and SCC-9 cancer cells shared some common features in the changes of m^6^A modification.

### Conjoint analysis of MeRIP-seq and RNA-seq data

**Figure 4:**
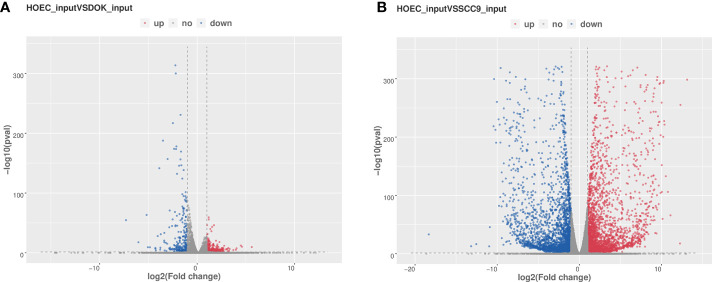
Volcano plots of differentially expressed genes in DOK and SCC-9 cells compared with HOEC cells. **(A)** HOEC VS DOK; **(B)** HOEC VS SCC-9. |log_2_FC|≥1.0 and *p* < 0.05.

We used RNA-seq (MeRIP-seq input library) data to analyze the difference of gene expression between any two cells. Compared with HOEC cells, DOK and SCC-9 cells had 328 and 3,531 significantly upregulated genes and 563 and 3,550 significantly downregulated genes, respectively (|log_2_FC|≥1.0 and *p* < 0.05; **Figure 4**), suggesting that SCC-9 cells have more obvious transcriptional changes than DOK cells. The top 20 (**Table 2**) or top 100 (**Figure S1**) genes differentially expressed between DOK and HOEC cells or between SCC-9 and HOEC cells also showed great differences, although many genes are related to the development and progression of tumors.

**Table 2:** The top 20 differentially expressed genes in DOK and SCC-9 cells compared with HOEC cells.

DOK				SCC-9			
Upregulated	log_2_FC	Downregulated	log_2_FC	Upregulated	log_2_FC	Downregulated	log_2_FC
AC004922	7.30	AC006064	-4.60	HDGFL3	11.19	CBLC	-13.12
IGFL2-AS1	5.18	RPPH1	-3.33	INA	11.05	FAR2	-12.31
CXCL8	3.87	SCARNA5	-3.28	SPINK13	10.43	CLDN3	-11.60
NMRAL2P	3.73	SCARNA10	-3.07	STC1	10.38	MAGED1	-11.40
SLC7A11	3.50	UPK3BL1	-2.78	COL4A5	10.33	HNF1A	-11.10
DHRS9	3.38	PRSS2	-2.52	LAMA4	10.27	RUBCNL	-11.04
AL137800	3.37	RNU1-3	-2.30	NNMT	10.27	FOXA3	-10.80
ZBED2	3.36	VWA5B2	-2.29	CPM	10.21	SMIM22	-10.78
TNFSF9	3.21	RN7SL3	-2.19	ANXA8	10.21	GALNT5	-10.73
DDIT4	3.03	MYL9	-2.04	XIST	10.21	PHGR1	-10.71

FPKM>2 in up-regulated genes.

**Figure 5:**
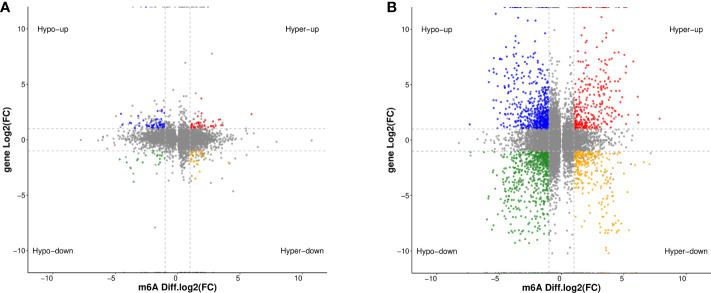
Distribution of differentially expressed genes with differential m^6^A peaks in DOK and SCC-9 cells, compared with HOEC cells. **(A)** HOEC VS DOK; **(B)** HOEC VS SCC-9. Hyper-up, m^6^A peak upregulated and mRNA expression upregulated; Hyper-down, m^6^A peak upregulated and mRNA expression downregulated; Hypo-up, m^6^A peak downregulated and mRNA expression upregulated; Hypo-down, m^6^A peak downregulated and mRNA expression downregulated.

**Figure 6:**
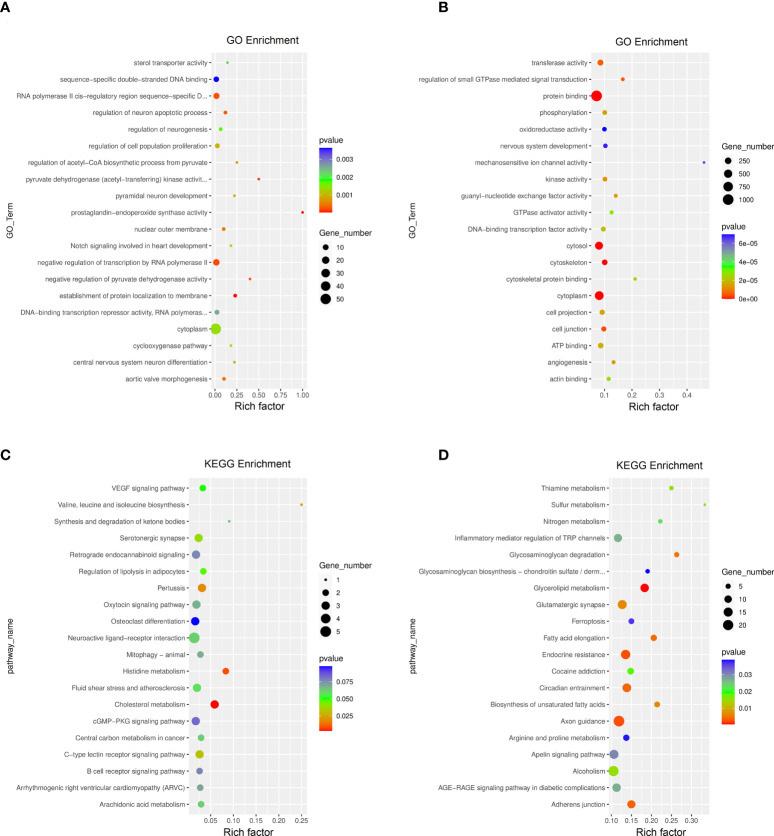
GO function and KEGG pathway enrichment of differentially expressed genes with differential m^6^A peaks. **(A, B)** Top 20 significantly enriched GO terms in DOK VS HOEC and SCC-9 VS HOEC cells, respectively. **(C, D)** Top 20 significant KEGG pathways in DOK VS HOEC and SCC-9 VS HOEC cells, respectively.

By jointly analyzing the MeRIP-seq and RNA-seq data, we further identified 354 differentially expressed mRNAs with differential m^6^A peaks in DOK cells, including 161 upregulated genes and 193 downregulated genes, compared with HOEC cells. These genes can be divided into four groups, including upregulated mRNAs with hypermethylated (hyper-up) or hypomethylated (hypo-up) m^6^A peaks and downregulated mRNAs with hypermethylated (hyper-down) or hypomethylated (hypo-down) m^6^A peaks (**Figure 5A**). This indicates that the expression changes of some genes are related to the changes of m^6^A modification in DOK cells. Obviously, there are more such genes in SCC-9 cells (**Figure 5B**), although the correlation between mRNA m^6^A methylation and its expression level needs to be further evaluated. The GO function and KEGG pathway enrichment of these genes was also analyzed (**Figure 6**).

Compared with HOEC cells, top 10 differentially expressed genes with differential m^6^A peaks in DOK cells or SCC-9 cells were summarized in **Table 3**. Although many of these genes are associated with tumors, they are not consistent in DOK and SCC-9 cells. Compared with DOK cells, the expression changes of differential genes in SCC-9 cells were also more significant. We noted that a transcript can have multiple differential m^6^A peaks, which can be hypermethylated or hypomethylated. The effects of different m^6^A modificaton on gene expression may be consistent or inconsistent. For example, TNFSF15, CPM and ENG all have hyper- and hypo- m^6^A peaks in a single transcript (**Table 3**), but they are all related to the upregulation of gene expression. ARHGEF26 has two hypo- m^6^A peaks and RGMB has three hypo- m^6^A peaks, which are all related to the downregulation of gene expression.

**Table 3:** The first 10 differentially expressed genes with differential m^6^A peaks in DOK cells or SCC-9 cells compared with HOEC cells.

DOK							
Hypo-up	log_2_FC	Hyper-up	log_2_FC	Hypo-down	log_2_FC	Hyper-down	log_2_FC
CXCL8	3.87	TNFSF15	2.57	EPHB3	-1.94	WFDC21P	-1.73
SLC7A11	3.50	UCA1	2.22	EDN1	-1.67	SNN	-1.59
ZBED2	3.36	NELL2	2.05	SNN	-1.59	GAL3ST2	-1.54
DDIT4	3.03	CALB2	2.04	ARHGEF26	-1.55	PBXIP1	-1.38
ANKRD37	2.87	ANGPTL4	1.95	RGMB	-1.36	PARS2	-1.35
TNFSF15	2.57	CD177	1.77	AC159540	-1.24	RHOV	-1.31
CXCL1	2.51	TM4SF1	1.70	CDHR1	-1.21	CBX6	-1.25
HMGCS2	2.36	SLC38A2	1.62	DIPK1A	-1.20	ZNF696	-1.25
TNFAIP3	2.27	BIRC3	1.57	IFITM10	-1.20	B3GNT9	-1.22
HMGCS1	2.22	IDI1	1.51	MECOM	-1.18	MYPOP	-1.22
**SCC-9**							
**Hypo-up**	**log_2_FC**	**Hyper-up**	**log_2_FC**	**Hypo-down**	**log_2_FC**	**Hyper-down**	**log_2_FC**
CPM	10.21	CPM	10.21	UTS2	-10.14	FAR2	-12.31
XIST	10.21	PDZD2	9.29	VIL1	-9.64	MAGED1	-11.40
SALL4	9.95	SMC1B	9.27	BICDL2	-9.46	RUBCNL	-11.04
FN1	9.71	WNT5A	9.08	KRT20	-9.25	SMIM22	-10.78
ARHGEF10	9.69	PSCA	9.04	PLEKHG6	-8.97	FUT2	-9.92
ENG	8.53	FSTL1	8.99	CYP3A5	-8.92	VIL1	-9.64
LOX	8.37	SHISAL1	8.92	MUC13	-8.81	MACC1	-9.38
GNB4	8.33	ENG	8.53	P2RY1	-8.39	TSTD1	-8.93
FERMT2	8.22	ACKR3	8.46	B3GNT3	-8.01	CYP3A5	-8.92
IGFBP7	8.17	LINC02593	8.44	GPX2	-7.88	PTPRH	-8.39

FPKM>2 in up-regulated genes.

### Expression of m^6^A regulatory genes

We wanted to know whether m^6^A methylation changes in DOK and SCC-9 cells were related to the regulatory genes of m^6^A modification. Firstly, we analyzed expression of key m^6^A regulators based on our sequencing data. Compared with HOEC cells, there was no significant change in the expression of m^6^A regulatory genes in DOK cells, but there was significant upregulation of FTO and IGF2BP1 and downregulation of METTL14 and IGF2BP2/3 in SCC-9 cells (|log_2_FC|≥1.0 and *p* < 0.05) (**Table S3**).

**Figure 7:**
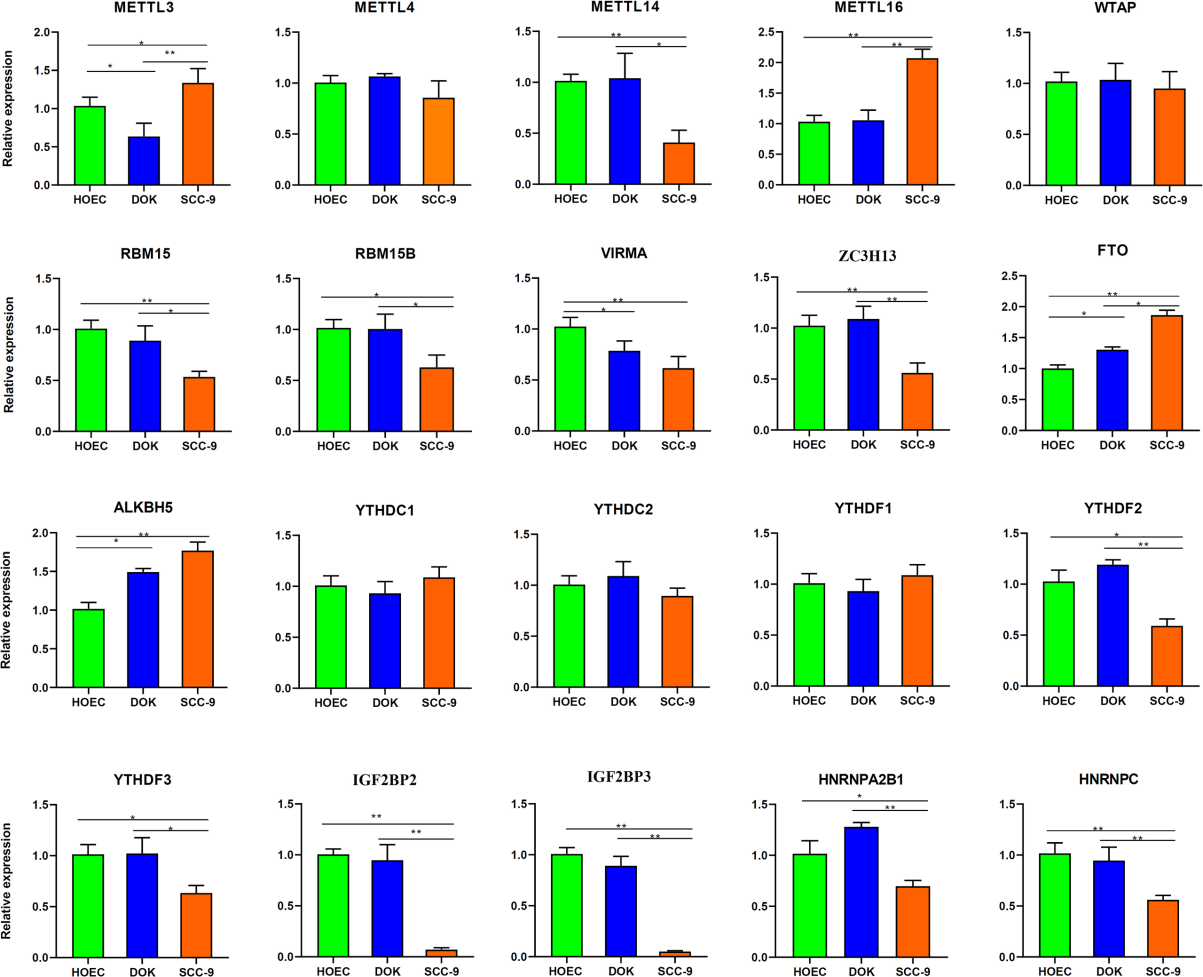
Expression of m^6^A regulatory genes in HOEC, DOK and SCC-9 cells detected by real-time RT-PCR. All bars show mean ± SD of three independent experiments. *p < 0.05, **p < 0.01.

We further determined the expression levels of these m^6^A regulatory genes in different cells by RT-PCR. Among the 20 genes detected, FTO and ALKBH5 were upregulated and METTL3 and VIRMA were downregulated in DOK cells compared with HOEC cells (0.01< *p* < 0.05, **Figure 7**). However, METTL16, FTO and ALKBH5 were significantly upregulated and METTL14, RBM15, VIRMA, ZC3H13, IGF2BP2/3 and HNRNPC were significantly downregulated in SCC-9 cells (*p* < 0.01, **Figure 7**). The expression of RBM15B, YTHDF2/3, HNRNPA2B1 and METTL3 in SCC-9 cells also decreased or increased to some extent (0.01< *p* < 0.05). The expression of most m^6^A regulatory genes was consistent with the sequence data (**Table S3**). Obviously, more m^6^A regulators in SCC-9 cells changed their expression level compared with DOK cells. These also accounted for the more extensive change in m^6^A modification in cancer cells compared with oral precancerous cells.

## Discussion

m^6^A methylation is the most common RNA post-transcriptional modification, which widely regulates a variety of cellular functions. Its significance in the development and progression of tumor has attracted extensive attention. In this study, through whole transcriptome m^6^A sequencing, we found that oral precancerous DOK cells showed, to some extent, changes of m^6^A modification compared with normal oral epithelial cells. Although the magnitude of these changes in DOK cells was less than that in SCC-9 cells, the two cell lines shared many GO terms and KEGG pathways, which were enriched by the mRNAs with m^6^A modification changes. A total of 354 differentially expressed mRNAs with differential m^6^A peaks were identified between DOK and HOEC cells. Among the 20 m^6^A regulatory genes detected by RT-PCR, FTO, ALKBH5, METTL3 and VIRMA in DOK cells were upregulated or downregulated to a certain extent, but in SCC-9 cells, the expression changes of 10 genes such as METTL14/16, FTO and IGF2BP2/3 were more significant. Our data suggest that precancerous cells do change their m^6^A modification status, but these changes may be more obvious in cancer cells than in precancerous cells.

At present, it is unclear whether there are changes of m^6^A modification in the precancerous stage, or whether m^6^A modification contributes to the initiation of cell transformation. Relevant research is very limited. In colorectal adenomas, a precancerous condition of colorectal cancer, expression of several m^6^A regulatory genes was found to be upregulated (28). However, the status of m^6^A modification and its relationship with m^6^A regulator expression have not been studied. Although the overall m^6^A modification was enhanced in precancerous oral submucosal fibrosis, the target genes and corresponding regulatory factors were unclear (29). In this study, we used a well-established precancerous cell line (30) as a model to investigate the potential contribution of m^6^A modification to the initiation of cell transformation. This is the first time to reveal the characteristics of m^6^A methylation in premalignant cells. It should be mentioned here that only one precancerous cell line was used in this study. This may only reflect the m^6^A modification characteristics of a single precancerous tissue. The genetic background of a single cell line is homogeneous, which is comparable with other cell lines. However, as immortalized cells, immortalization may change some characteristics of the original cells, resulting in false reflection of m^6^A modification state. In addition to analyzing more cell lines, we can also directly analyze m^6^A modification of some oral precancerous tissues, such as lichen planus, leukoplakia and erythroplakia (31, 46). Since it is usually difficult to obtain enough oral precancerous tissue samples, other similar tissues with precancerous characteristics, such as cervical squamous intraepithelial lesions (31) and colorectal adenomas, can also be selected.

We noted that many differentially expressed genes with differential m^6^A peaks in DOK cells (**Table 3**) are closely related to tumorigenesis. For example, E2F1 transcriptional activation of neural epidermal growth factor-like 2 (NELL2) accelerates the progression of non-small cell lung cancer (47). Calbindin 2 (CALB2) promotes metastasis of hepatocellular carcinoma (48). The upregulation of transmembrane 4 L six family member 1 (TM4SF1) in papillary thyroid carcinoma patients is associated with lymph node metastases (49). Therefore, the role of the m^6^A target genes identified in tumorigenesis is worth studying. As mentioned above, many m^6^A regulators are also associated with tumorigenesis and progression. In this study we also found that the expression of several m^6^A regulators changed in oral precancerous cells. Their exact function in tumors remains to be evaluated.

Compare with DOK cells, oral squamous cell carcinoma cells have more extensive changes in m^6^A modification, involving more m^6^A peaks and more genes. On the one hand, this suggests that the epigenetic modification of m^6^A may play an important role in carcinogenesis. On the other hand, this may indicate that there is still a long way to go from precancerous lesions to cancer. We even found that the mRNA of the same gene can have different m^6^A modifications in precancerous cells and cancer cells (**Table 1**). For example, eukaryotic translation initiation factor 3 subunit C (EIF3C), a subunit of the protein translation initiation factor EIF3, was hyper-methylated in exonic region in DOK cells and 3`UTR in SCC-9 cells (**Figure 2**). It was reported that m^6^A reader YTHDF1 bound to m^6^A-modified EIF3C mRNA and promoted the translation of EIF3C and the overall translational output, consequently facilitating tumorigenesis and metastasis of ovarian cancer (50). Compared with cancer cells, the change of m^6^A regulatory gene expression in precancerous cells also seems to be less obvious. It can be supposed that the regulatory genes in precancerous cells are mainly regulated by changing enzyme activity rather than inducing gene expression. This needs further research to evaluate. Therefore, we must explore the function and significance of the key m^6^A target genes and regulators identified in this study in tumorigenesis. This may provide potential intervention targets for the prevention of cancer development through epigenetic modification in the future.

## Data availability statement

The data presented in the study are deposited in the GEO repository, accession number GSE213714.

## Author contributions

DY, WZ and SC contributed conception and design of the study. XC, LC, YT, YH, KP, LY, WX collected samples and performed experiments. All authors participated in the writing of the manuscript and confirmed the final review of the manuscript.

## Funding

This study was funded by the National Natural Science Foundation of China (No. 81873711 and No. 31670788) and by the Open Fund of Guangdong Key Laboratory of Pharmaceutical Functional Genes (No. 2020B1212060031 and No. 2017B030314021).

## Conflict of interest

The authors declare that the research was conducted in the absence of any commercial or financial relationships that could be construed as a potential conflict of interest.

The handling editor ZL declared a shared parent affiliation with the authors at the time of review.

## Publisher’s note

All claims expressed in this article are solely those of the authors and do not necessarily represent those of their affiliated organizations, or those of the publisher, the editors and the reviewers. Any product that may be evaluated in this article, or claim that may be made by its manufacturer, is not guaranteed or endorsed by the publisher.

## Supplementary material

Supplementary Figure 1 Hierarchical clustering heatmap of the top 100 differentially expressed genes in DOK and SCC-9 cells compared with HOEC cells. **(A)** HOEC VS DOK; **(B)** HOEC VS SCC-9.
