# Supplementary figures and images for: Transcriptome-wide m6A methylome analysis uncovered the changes of m6A modification in oral pre-malignant cells compared with normal oral epithelial cells
Source: Front Oncol. 2022 Sep 28;12:939449. doi: 10.3389/fonc.2022.939449 (PMC9554554; doi:10.3389/fonc.2022.939449)

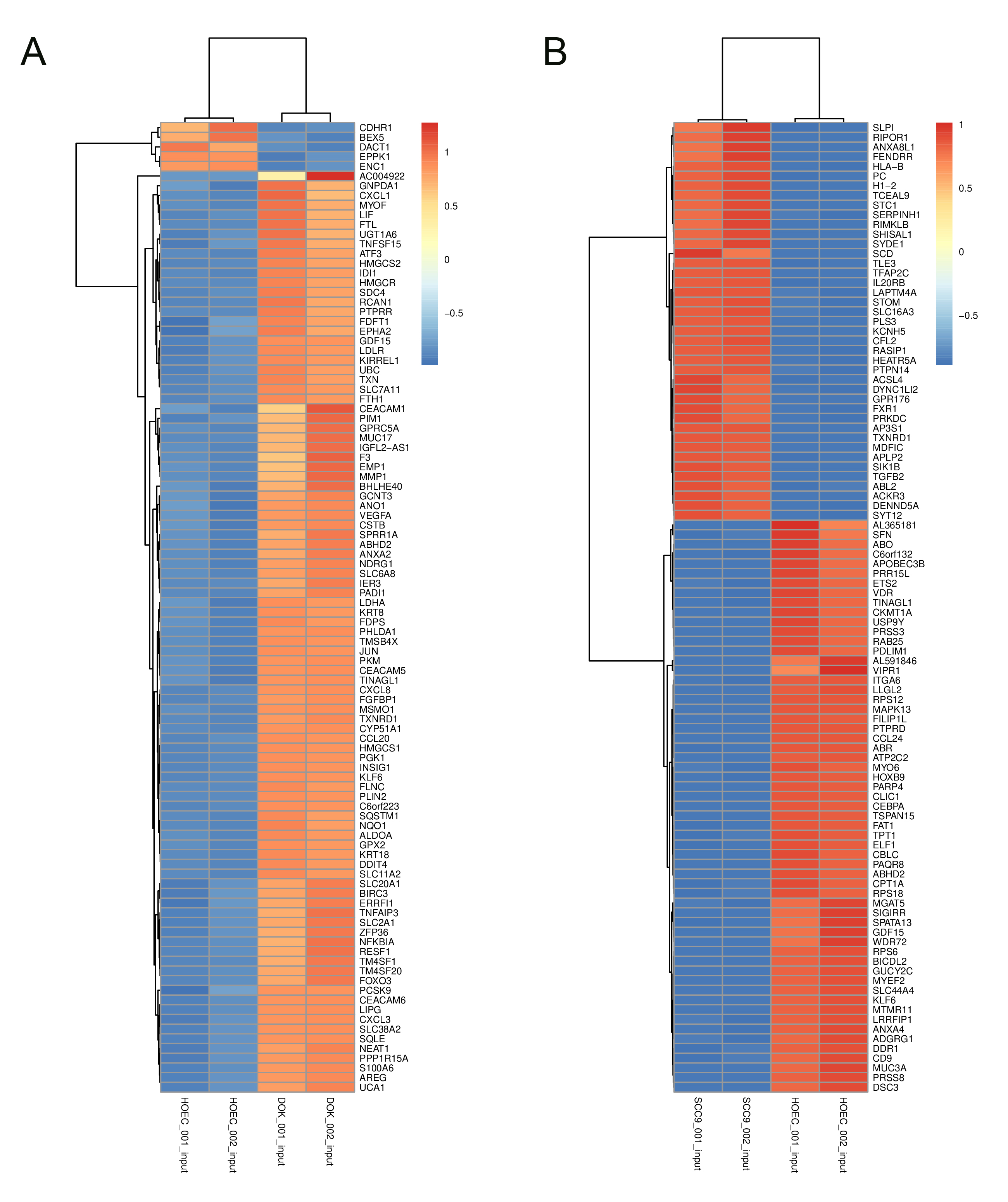

Supplement: Supplementary Figure 1 — Hierarchical clustering heatmap of the top 100 differentially expressed genes in DOK and SCC-9 cells compared with HOEC cells. (A) HOEC VS DOK; (B) HOEC VS SCC-9. [file Image_1.jpeg]
