# Supplementary material for: Transcriptome-wide m6A methylome analysis uncovered the changes of m6A modification in oral pre-malignant cells compared with normal oral epithelial cells
Source: Front Oncol. 2022 Sep 28;12:939449. doi: 10.3389/fonc.2022.939449 (PMC9554554; doi:10.3389/fonc.2022.939449)
Supplement: Supplementary file 2 [file Table_1.docx]

Table S1. The primers used for real-time RT-PCR

| Genes | Forward | Reverse |
| --- | --- | --- |
| METTL3 | CAAGCTGCACTTCAGACGAA | GCTTGGCGTGTGGTCTTT |
| METTL4 | TATCCCTCTTGGTCTGTGGAG | ACCTTCGTAGGGCTTTTTGTG |
| METTL14 | AGAAACTTGCAGGGCTTCCT | TCTTCTTCATATGGCAAATTTTCTT |
| METTL16 | CTCTGACGTGTACTCTCCTAAGG | TACCAGCCATTCAAGGTTGCT |
| WTAP | GGCGAAGTGTCGAATGCT | CCAACTGCTGGCGTGTCT |
| RBM15 | GGCTGCCTGAGGAGAGTGGAG | CGGCTACTGCTCAATTCTGGACTG |
| RBM15B | ATCTTTCAGAGTACGCTCAGAC | CTAGGATATGCATAGACGTGGG |
| VIRMA | GCAACTTCAGGCATTAAGTTCA | GTATTGCCTTGTCGAATCTGTC |
| ZC3H13 | GATCAGTTAAAGCGTGGAGAAC | CTCTCTGTCGTGTTCATATCGA |
| FTO | ACTTGGCTCCCTTATCTGACC | TGTGCAGTGTGAGAAAGGCTT |
| ALKBH5 | CGGCGAAGGCTACACTTACG | CCACCAGCTTTTGGATCACCA |
| YTHDC1 | AGATGGGTCTGTCAGATCTGGT | TCTGAACCTGCATATGACTCTGAT |
| YTHDC2 | GAGAATTGGGCTGTCGTTAAAG | TGAAGCAGGATGAAATCGTACT |
| YTHDF1 | CAAGCACACAACCTCCATCTTCG | GTAAGAAACTGGTTCGCCCTCAT |
| YTHDF2 | ACTTCTCAGCATGGGGAAATAA | TATTCATGCCAGGAGCCTTATT |
| YTHDF3 | TCAACCACCACAACCACAGCAG | TGAAGCACTGACAGGTACAACACC |
| IGF2BP2 | GATGAACAAGCTTTACATCGGG | GATTTTCCCATGCAATTCCACT |
| IGF2BP3 | GAGGCGCTTTCAGGTAAAATAG | AATGAGGCGGGATATTTCGTAT |
| HNRNPA2B1 | TGGAGGTAGCCCCGGTTATG | GGACCGTAGTTAGAAGGTTGCT |
| HNRNPC | ACAGATCCTCGCTCCATGAACTCC | TTCTGCCATCCTCTCCTGCTACAG |
| β-actin | CTTAGTTGCGTTACACCCTTTCTTG | CTGTCACCTTCACCGTTCCAGTTT |
