# Supplementary material for: Transcriptome-wide m6A methylome analysis uncovered the changes of m6A modification in oral pre-malignant cells compared with normal oral epithelial cells
Source: Front Oncol. 2022 Sep 28;12:939449. doi: 10.3389/fonc.2022.939449 (PMC9554554; doi:10.3389/fonc.2022.939449)
Supplement: Supplementary file 3 [file Table_2.docx]

Table S2. Summary of sequencing data and reads alignment statistics from MeRIP-seq and RNA-seq of HOEC, DOK, and SCC-9 cells

| Sample_ID | Raw_reads | Raw_bases | Valid_reads | Valid_bases | Valid% | Q20% | Q30% | GC% |
| --- | --- | --- | --- | --- | --- | --- | --- | --- |
| HOEC_1_IP | 41657098 | 6.29G | 40105776 | 5.44G | 86.44 | 98.42 | 95.35 | 50.22 |
| HOEC_2_IP | 37700682 | 5.69G | 36375534 | 4.97G | 87.38 | 98.40 | 95.31 | 50.34 |
| DOK_1_IP | 40359674 | 6.09G | 38812338 | 5.27G | 86.51 | 98.43 | 95.38 | 50.34 |
| DOK_2_IP | 44157610 | 6.67G | 42618566 | 5.80G | 87.05 | 98.39 | 95.28 | 50.32 |
| SCC-9_1_IP | 41278778 | 6.23G | 39431982 | 5.37G | 86.15 | 98.37 | 95.29 | 50.34 |
| SCC-9_2_IP | 44250990 | 6.68G | 42428028 | 5.80G | 86.76 | 98.37 | 95.30 | 50.56 |
| HOEC_1_input | 46624842 | 7.04G | 45224674 | 6.22G | 88.28 | 98.47 | 95.36 | 48.96 |
| HOEC_2_input | 50879172 | 7.68G | 49369474 | 6.78G | 88.23 | 98.49 | 95.40 | 48.96 |
| DOK_1_input | 48365388 | 7.30G | 46905062 | 6.45G | 88.30 | 98.49 | 95.40 | 49.01 |
| DOK_2_input | 47458110 | 7.17G | 45955372 | 6.32G | 88.14 | 98.46 | 95.34 | 48.99 |
| SCC-9_1_input | 49860040 | 7.53G | 48291204 | 6.65G | 88.29 | 98.46 | 95.40 | 49.72 |
| SCC-9_2_input | 50625142 | 7.64G | 48993602 | 6.75G | 88.24 | 98.47 | 95.44 | 49.89 |
