# Supplementary material for: Transcriptome-wide m6A methylome analysis uncovered the changes of m6A modification in oral pre-malignant cells compared with normal oral epithelial cells
Source: Front Oncol. 2022 Sep 28;12:939449. doi: 10.3389/fonc.2022.939449 (PMC9554554; doi:10.3389/fonc.2022.939449)
Supplement: Supplementary file 4 [file Table_3.docx]

Table S3. The expreesion of m^6^A regulatory genes including m^6^A writers, erasers and readers in DOK and SCC-9 cells.

|  | DOK |  | SCC-9 |  |
| --- | --- | --- | --- | --- |
| Genes | Expression | log_2_FC | Expression | log_2_FC |
| METTL3 | down | -0.06 | down | -0.20 |
| METTL14 | down | -0.07 | down | -1.09 |
| METTL16 | up | 0.08 | up | 0.94 |
| WTAP | up | 0.02 | up | 0.42 |
| RBM15 | up | 0.31 | down | -0.12 |
| RBM15B | down | -0.32 | down | -0.12 |
| VIRMA | down | -0.23 | down | -0.13 |
| ZC3H13 | down | -0.03 | down | -0.74 |
| FTO | down | -0.13 | up | 1.74 |
| ALKBH3 | down | -0.27 | down | -0.07 |
| ALKBH5 | down | -0.02 | up | 0.56 |
| YTHDC1 | up | 0.07 | up | 0.09 |
| YTHDC2 | down | -0.11 | up | 0.31 |
| YTHDF1 | down | -0.29 | up | 0.36 |
| YTHDF2 | down | -0.12 | down | -0.85 |
| YTHDF3 | - | 0.00 | down | -0.27 |
| IGF2BP1 | down | -0.56 | up | 7.18 |
| IGF2BP2 | down | -0.04 | down | -3.91 |
| IGF2BP3 | down | -0.05 | down | -3.67 |
| HNRNPA2B1 | down | -0.07 | up | 0.17 |
| HNRNPC | up | 0.01 | down | -0.11 |
| eIF3B | up | 0.03 | down | -0.01 |
